# Antimicrobial Resistance of *Neisseria gonorrhoeae* Isolates among Men Who Have Sex with Men in Lower Silesia, Poland

**DOI:** 10.3390/pathogens13100890

**Published:** 2024-10-11

**Authors:** Martyna Biała, Beata Mączyńska, Konrad Starzyński, Danuta Rurańska-Smutnicka, Anna Secewicz, Paulina Szuba, Bartosz Szetela

**Affiliations:** 1 Department of Infectious Diseases, Liver Diseases and Acquired Immune Deficiences, Wroclaw Medical University, 51-149 Wroclaw, Poland; 2 Department of Pharmaceutical Microbiology and Parasitology, Wroclaw Medical University, 50-556 Wroclaw, Poland; 3 All Saints’ Clinic, Wroclawskie Centrum Zdrowia SP ZOZ, 50-136 Wroclaw, Poland

**Keywords:** *Neisseria gonorrhoeae*, MSM, antibiotic resistance, STI

## Abstract

*Neisseria gonorrhoeae* (NG) has developed resistance to nearly all antibiotics used for its treatment. However, very limited data are available regarding the antimicrobial resistance of NG isolates among MSM in Poland. The aim of this study was to evaluate the susceptibility of *Neisseria gonorrhoeae* isolates in this key population. We investigated the antimicrobial susceptibility of NG isolates to six antimicrobials (ceftriaxone, cefixime, azithromycin, ciprofloxacin, tetracycline, and benzylpenicillin). Minimum inhibitory concentrations (MICs; mg/L) were determined using Etests on gonococcal isolates. One hundred high-risk MSM were included in the study (25 HIV-positive and 75 HIV-negative using pre-exposure prophylaxis for HIV). The rate of NG infection was 28%. All NG isolates were susceptible to cefixime and ceftriaxone. Susceptibility to azithromycin was found in 69.2% (18/26) of the NG isolates and resistance in 30.8% (8/26) of NG isolates. Susceptibility to tetracycline was found in 50% (13/26) of the isolates and resistance in 50% (13/26) of the isolates. We observed gonorrhea to be more prevalent in patients with a higher number of oral sexual contacts. Increasing azithromycin resistance is especially concerning for future treatment options, especially if ceftriaxone/cefixime resistance starts to develop and for people with beta-lactam antibiotics allergies. Doxy-PEP might lose its partial efficacy for NG soon.

## 1. Introduction

Gonorrhea is a common sexually transmitted infection caused by *Neisseria gonorrhoeae* (NG). Rates of NG infections are the highest among key populations such as men having sex with men, sex workers, transgender women, adolescents, and young people [1]. Risk factors for infection include multiple sex partners, casual sex partners, group sex, and chemsex [2]. Almost 90% of bacterial STIs among MSM, especially at oropharyngeal and rectal sites [3,4,5], are asymptomatic, making diagnosis more difficult [6]. Therefore, there are clear recommendations for screening every 3–6 months, including three-site testing for *Chlamydia trachomatis* and *Neisseria gonorrhoeae* as well as HIV, HCV, and syphilis in key populations [7,8,9]. Kissing, oral sex without condoms, and saliva being used as lubricant may be responsible for the easy transmission of NG. Even individuals with stable circuits of partners may be at an increased risk of NG infection and may introduce NG to their sexual groups [10]. However, regular NG and CT screening and treatment of asymptomatic patients in dense transmission networks might lead to an increase in resistance rather than a decrease [6,11]. There is also increasing evidence that antimicrobial use could be contributing to antimicrobial resistance, and data indicate that drug-resistant NG might be an increasing public health problem. NG has even been declared as a ‘high’-priority pathogen by the WHO in terms of research and development for new antibiotics [12].

The objectives of this study were to analyze the susceptibility of NG isolates from MSM to penicillin, cefixime, ceftriaxone, tetracycline, ciprofloxacin, and azithromycin.

## 2. Materials and Methods

MSM living with HIV (PLWH) (either naïve or already on combined antiretroviral therapy—cART) and MSM PrEP users were invited to take part in this study. Inclusion criteria were age >18 years, being an MSM, having had multiple partners in the last 3 months, or having had sexual contact with a partner with confirmed NG infection. Both asymptomatic and symptomatic patients were included. Symptoms suggesting NG included a burning sensation during urination, discharge from the urethra, painful or swollen testicles, discharge from the anus, anal itching, anal soreness, anal bleeding and painful bowel movement, swollen lymph nodes, a burning feeling in the throat, and a sore or dry throat. Upon inclusion in the study, a self-applied online questionnaire (tablet available onsite) was conducted to collect sociodemographic data, the number of sexual partners in the last three months, the type of sexual intercourse and number of times having had sexual intercourse (oral/anal), condom use depending on the type of sex, chemsex, the use of substances (alcohol, cigarettes, drugs), PrEP use, the presence of other sexually transmitted infections (including syphilis, gonorrhea, chlamydia, HCV), and doxy-PEP use.

NG isolates were obtained from oropharyngeal, urethral, and rectal swabs from MSM attending an outpatient HIV clinic—All Saint’s Clinic in Wroclaw (Poradnia Wszystkich Swietych, Wroclawskie Centrum Zdrowia)—between October 2022 and December 2023. We used Amies with Charcoal Transport Medium for swabs. NG swabs were tested at the Department of Pharmaceutical Microbiology and Parasitology, Wroclaw Medical University, Poland. Swabs were inoculated in Thayer Martin, GC, and chocolate agar culture mediums and incubated in a 5% CO_2_ atmosphere. The identification of *N. gonorrhoeae* was performed according to the API NH test (bioMerieux, Marcy-l’Etoile, France). Confirmed isolates of *N. gonorrhoeae* were stored at −80 °C. We investigated the antimicrobial susceptibility of the isolates to six antimicrobials (ceftriaxone, cefixime, azithromycin, ciprofloxacin, tetracycline, and benzylpenicillin). Minimum inhibitory concentrations (MICs; mg/L) were determined using Etests (bioMerieux, Marcy-l’Etoile, France). The Etest method was performed on Mueller–Hinton Agar (with 5% defibrinated horse blood and NAD) and supplemented GC. The results obtained were analyzed in accordance with the clinical breakpoints set by the European Committee on Antimicrobial Susceptibility Testing (EUCAST; version 14.0).

The statistical analysis were conducted using EPIINFO Ver. 7.2.3.1 and Statistica Ver. 13.3. For all groups, the number of cases (N) and the mean (X), median (M), range (min–max), lower and upper quartile (25q–75q), and standard deviation (SD) of the parameters were calculated. Median (interquartile range [IQR]) was used for the description of non-normally distributed data. Qualitative variables were presented as absolute values and percentages (%). The normality assumption was assessed by carrying out the Shapiro–Wilk test for the groups of data. Levene′s test was carried out to assess the homogeneity of variances assumption. For qualitative parameters, the frequency of the traits or characteristics in groups was analyzed using the χ^2^_df_ test with the appropriate number of degrees of freedom df (df = (m − 1) × (*n* − 1), where m—number of rows, *n*—number of columns). The verification of the hypothesis of equality of mean groups of heterogeneous variance was performed by the non-parametric Mann–Whitney U test. *p* ≤ 0.05 was considered statistically significant.

## 3. Results

The study included 100 MSM (25 PLWH and 75 HIV PrEP users (Table 1)). In total, 65% (16/25) of the PLWH and 53% (40/75) of the PrEP users were symptomatic (*p* = 0.38). The age distribution in the studied groups was similar, and the median age was 34.0 years [interquartile range (IQR) 30–38 years]. Most patients (83%) lived in large cities (over 500,000 people). The median number of sexual partners in the last three months was five and did not differ between the groups (*p* = 0.88). Overall, 94 out of 100 MSM declared sexual contact with men only and six declared sexual contact with both men and women. In total, 64% of all MSM declared that they had had sexual contact only with Polish partners, while 36% declared that they had had sexual contact with other nationalities (the most common were Spanish, German, and British). A total of 89% reported they had a sexually transmitted infection (STI) diagnosed in the past (syphilis, gonorrhea, or chlamydia), 8% had no history of STIs, while 3% could not remember. No difference in past STI prevalence was seen between the two groups (*p* = 0.24). In total, 18% of all patients had ever taken doxy-PEP, 66% declared that they had never used it, and 16% did not know what it was. MSM living with HIV used doxy-PEP more often than PrEP users (*p* = 0.015). Chemsex was reported by 54% of patients, with no difference between the two groups (*p* = 0.17). Very few patients used condoms during anal sex, and even fewer during oral sex in the last month, confirmed by the median being 0% in both cases.

### 3.1. Microbiological Data

The NG infection rate was 28%. Positive cultures from urethral sites predominated (*n* = 18), followed by rectal (*n* = 8) and oropharyngeal (*n* = 2). Differences between PLWH and PrEP users are shown in Table 2 (*p* = 0.26).

Patients with NG infections had a higher number of oral contacts in the last month (median 8, IQR 3–10) in comparison to patients without NG infections (median 4, IQR 2–10) (*p* = 0.0472).

There were no statistically significant differences between NG-positive cultures and other variables included in Table 1 (e.g., number of sexual partners, number of anal contacts in the last month).

Eight positive cultures were from asymptomatic patients and included two urethral, two oropharyngeal, and four rectal ones.

### 3.2. Antimicrobial Resistance

Two isolates were excluded from the analysis as there was no growth during susceptibility testing. The most common resistance was seen for ciprofloxacin, with only 11.5% of isolates susceptible. Azithromycin resistance was found in 30.8% of the isolates, leaving 69.2% susceptible. Tetracycline resistance was seen in 50% of isolates. Susceptibility to penicillin was found only in 26.9%. All isolates were susceptible to cefixime and ceftriaxone—see details in Table 3.

## 4. Discussion

There is a scarcity of microbiological resistance pattern reports for the Eastern European region and almost no longitudinal monitoring, even among the most impacted populations. This is the first prospective cohort monitoring the antibiotic resistance profiles of NG among high-risk MSM in Poland. NG antibiotic resistance reports from Poland are very limited. According to the Euro-GASP 2020 genomic survey, resistance to azithromycin and ciprofloxacin was observed in 35% (7/20) and 50% (10/20) of NG isolates in Poland, respectively [13]. Furthermore, all of these NG isolates (20/20) were susceptible to ceftriaxone and cefixime [13]. Moreover, the *Gonococcal antimicrobial susceptibility surveillance* summary of results for 2022 indicated that 40% (6/15) and 66.7% (10/15) of NG isolates in Poland were resistant to azithromycin and ciprofloxacin, respectively [14]. No resistance to cephalosporin was detected in Poland [14]. We have shown that resistance profiles in our cohort follow European trends, with no or low-level cephalosporin resistance and with high-level fluoroquinolone and azithromycin resistance already being rather widespread. The level of resistance to azithromycin of 30.8% as well as the level of resistance to ciprofloxacin of 88.5% are higher than in most European countries [14]. There are also inter-country differences most probably reflecting local prescription patterns and antibiotic policy. These differences might be the result of either actual changes in resistance, regional differences, or more prevalent resistance among MSM who use antibiotics more often to treat STIs. Wong et al. reported that over 50% of empirical ceftriaxone therapies for anorectal symptoms were unnecessary, as resulting nucleic acid amplification assays yielded negative results both for NG and *Chlamydia trachomatis* (CT) [15]. With limited access to healthcare providers who can correctly diagnose and treat STIs in Poland, patients report using antibiotics from home or from colleagues or medical professionals offering incorrect empirical antibiotics—fluoroquinolones are still commonly used, despite an existing alert and cautions that this group of antibiotics may increase the risk of serious side effects [16,17]. Furthermore, data showed that commensal *Neisseria* species (e.g., *N. subflava*, *N. mucosa*) of the oropharynx represent a significant reservoir of antimicrobial resistance determinants that can be transferred to NG in populations where pharyngeal gonorrhea is quite common [18]. Van Dijk et al. have shown an increased number of antibiotic-resistant genes among MSM on PrEP, and they can easily be taken up by NG [19]. This might also be a potential explanation for the resistance profile of NG, especially when taking into account oral contacts as the most important risk factor of infection in key populations.

Common resistance to tetracycline has been reported worldwide; thus, doxy-PEP will not be as effective for NG as for CT or *Treponema pallidum*. There has been a huge amount of interest in doxy-PEP among MSM from the very beginning, even before efficacy trials results had been published [20]. Knowing that we can expect the increased consumption of doxycycline, dosed correctly or not, its efficacy most probably will decrease over time as even more NG isolates become resistant. In the *ANRS 174 DOXYVAC* trial, there was already a significant decrease in efficacy from 50% in 2022 to 33% in 2023 for NG, with unchanged syphilis and a CT incidence reduction of 83% [21].

There has been no patient reported to have cefixime or ceftriaxone resistance; however, according to the WHO, NG antimicrobial resistance has risen rapidly in recent years and has led to reduced therapeutic options, especially in wealthier countries, but this might be only the result of better access to testing [22]. The rising prevalence of azithromycin resistance is especially concerning for future treatment efficacy, especially if ceftriaxone resistance starts to spread more widely [23].

## 5. Conclusions

All isolates remain susceptible to cephalosporins. Increasing azithromycin resistance is especially concerning for future treatment options, especially if ceftriaxone/cefixime resistance starts to develop and for people with beta-lactam antibiotic allergies. Resistance to azithromycin in 30% of isolates should prompt changes in the combined treatment guidelines for NG.

Bacterial cultures remain an important diagnostic method for the prospective follow-up of antimicrobial susceptibility patterns in NG and allow emerging changes to be spotted at the population level, as well as clustering. A system offering easy access to testing for populations at risk is needed, including the monitoring of regional resistance patterns, their dynamics, and the spread of resistant strains. Moreover, there is a need for strategies to promote rational antibiotic use in STI treatment.

MSM remain at increased risk of NG transmission, especially when having a higher number of oral contacts. Doxy-PEP’s partial efficacy for NG might decrease soon, as a rapid increase in doxycycline resistance in isolates from this group of patients is probable.

### Limitations

Our cohort is probably not representative of the whole population, as only MSM were included. Due to the relatively small sample size and single-site recruitment, resistance patterns might differ in other regions.

## Figures and Tables

**Table 1:** Group characteristics.

	PrEP	PLWH	*p*
Number of patients	75/100	25/100	NA
Age [years], median [IQR]	34 (28–38)	34.5 (32–38)	0.68
Exclusively MSM	93.33% (70/75)	95.83% (23/24)	0.66
Bisexual patients	6.67% (5/75)	4.17% (1/24)	0.66
Polish partners only	61.33% (46/75)	75% (18/24)	0.22
History of STIs	86.67% (65/75)	95.83% (23/24)	0.24
Doxy-PEP use	12% (9/75)	37.5% (9/24)	0.015
Chemsex participation	50.67% (38/75)	66.67% (16/24)	0.17
No of sexual partners in the last 3 months, median, [IQR]	6 (3–12)	5 (3.5–10)	0.88
No of oral sexual contacts in the last month, median, [IQR]	5 (3–10)	3 (2–10)	0.33
No of anal sexual contacts in the last month, median, [IQR]	4 (2–8)	3 (2–5)	0.37
Symptomatic infections	53.3% (40/75)	64% (16/25)	0.38
Positive NG cultures	28% (21/75)	28% (7/25)	0.26

**Table 2:** NG isolates in PLWH and PrEP users.

	Urethral [*n*]	Oropharyngeal [*n*]	Rectal [*n*]
PrEP user	15	2	4
PLWH	3	0	4
Total	18	2	8

**Table 3:** Antimicrobial susceptibility of NG isolates.

	Penicillin		Cefixime		Ceftriaxone		Ciprofloxacin		Tetracycline		Azithromycin	
	Isolates [*n*]	%	Isolates [*n*]	%	Isolates [*n*]	%	Isolates [*n*]	%	Isolates [*n*]	%	Isolates [*n*]	%
S—susceptible	7	26.9%	26	100.0%	26	100.0%	3	11.5%	13	50.0%	18	69.2%
I—intermediate	11	42.3%		0.0%		0.0%	2	7.7%		0.0%		0.0%
R—resistant	8	30.8%		0.0%		0.0%	21	80.8%	13	50.0%	8	30.8%
*n* = 26	26	100.0%	26	100.0%	26	100.0%	26	100.0%	26	100.0%	26	100.0%

## Data Availability

The data presented in this study are available and can be shared upon reasonable request sent to the corresponding author.
